# Prenatal Valproic Acid Exposure Affects Song Learning in Zebra Finches: A Potential Model for Vocal Development in Autism

**DOI:** 10.3390/life15071058

**Published:** 2025-07-01

**Authors:** Estifanos Ghebrihiwet Tewelde, Boglárka Morvai, Gergely Zachar, Ákos Pogány

**Affiliations:** 1 Department of Ethology, Eötvös Loránd University, 1117 Budapest, Hungary; estifanosghebrihiwet@gmail.com (E.G.T.); boglarka.morvai@ttk.elte.hu (B.M.); akos.pogany@ttk.elte.hu (Á.P.); 2 Department of Biology, Mai-Nefhi College of Science, Mai-Nefhi P.O. Box 12676, Eritrea; 3 Department of Anatomy, Histology and Embryology, Semmelweis University, 1094 Budapest, Hungary

**Keywords:** vocal learning, acoustic communication, neurodevelopmental disorder, animal model of ASD, zebra finch, songbird, prenatal valproic acid (VPA), autism spectrum disorder (ASD)

## Abstract

Autism spectrum disorder (ASD) encompasses a range of neurodevelopmental conditions characterized by impairments in social abilities and communication. Studying appropriate animal models can enhance our understanding of the neural mechanisms underlying these conditions, potentially leading to improved treatment and intervention strategies. Modeling impairments in linguistic development and vocal communication caused by autism had been a challenging goal for a long time. Prenatal valproic acid (VPA) treatment has been successfully used to induce ASD-like behavioral symptoms in several vertebrate species including birds. Applying VPA-treatment on songbirds, therefore, offers a promising research paradigm to model ASD. In this study, we investigated the effect of embryonic VPA treatment on song learning in the highly social zebra finch (*Taeniopygia guttata*). Fertilized eggs were injected with either 0.45 µmol VPA or a saline solution on day 9 of incubation, and hatchlings were allowed to stay with their parents until day 35 post-hatching to facilitate song learning from the father. Once male offsprings reached adulthood, their songs were recorded and compared to those of their fathers. VPA-exposed males exhibited significantly greater similarity to their fathers’ songs compared to control males (74% vs. 31%, respectively), suggesting a reduced ability to modify and develop their own vocal patterns as subadults. Additionally, they showed higher entropy in their songs compared to controls (−1.4 vs. −1.7), indicating more disorganized vocalizations. These findings suggest that prenatal VPA exposure disrupts typical song learning and vocal development in zebra finches, likely by affecting neural mechanisms involved in vocal learning and crystallization of the songs. Our study suggests VPA treatment in songbirds provides a useful tool to model and investigate linguistic developmental disorders related to ASD in humans.

## 1. Introduction

Autism spectrum disorder (ASD), ranked as the third most common human developmental disorder, encompasses a range of neurodevelopmental conditions characterized by difficulties in social interaction, communication, and behavior [1]. Besides a lack of interest in others, further symptoms include difficulties in expressing emotions, resistance to physical contact, and repetitive behaviors [1,2]. Classic autism, at one end of the spectrum, is also associated with delayed or absent speech development and, in some cases, cognitive delays [3,4]. The causes of ASD remain unclear, though research suggests a complex interplay of genetic, biological, psychosocial, and environmental risk factors [1,5].

Valproic acid (VPA), chemically known as 2-propyl-pentanoic acid, is a branched short-chain fatty acid derived from valeric acid, a compound found in the Valerian plant (*Valeriana officinalis*). It exhibits significant pharmacological properties and has been widely prescribed to treat epilepsy, bipolar, and anxiety disorders [6,7,8,9,10]. At non-toxic therapeutic doses, it acts as a potent and selective inhibitor of histone deacetylase (HDAC) enzymes [6]. However, VPA also induces teratogenic effects, particularly in human embryos, leading to the ‘valproate syndrome’, which is characterized by reduced verbal intelligence and communication difficulties commonly associated with ASD [11].

VPA’s teratogenic effects have been documented in various species and taxa, including mammals, birds, and fish. In domestic chickens, exposure to VPA reduces hatching rates, and hatched chicks display paralysis, locomotor impairments, and altered social behaviors [12,13]. In mice, prenatal VPA exposure results in delayed physical growth, reduced play behavior, and abnormal social interactions in juveniles, which persists into adulthood [2,14]. Similarly, zebrafish embryos exposed to VPA exhibit developmental abnormalities, including skeletal deformities, abnormal swimming patterns, and pericardial fluid accumulation. These impairments affect locomotion, social interactions, and macrocephalic traits linked to ASD. The severity of these symptoms depends on VPA concentration and timing of exposure [15,16,17]. Embryonic VPA treatment is proven to be useful as a pharmacological model of autism in rodents [18,19], domestic chicks [20], and zebrafish [21]. Certain aspects of autism, however, are very difficult if not impossible to study in the aforementioned N taxa, including the impairment of linguistic development which occurs in autistic children [22].

The zebra finch (*Taeniopygia guttata*), a highly social songbird, is an ideal species for studying developmental impairments of social behavior and vocal communication. These passerine exhibit sexual dimorphism and form monogamous pair bonds following mate choice based partially on courtship songs [23,24]. Parents cooperate to raise their offspring [25], and behavioral coordination during biparental care relies on vocalizations [26]. Zebra finches are well-suited for research due to their adaptability; they breed year-round and well in the laboratory with a generational cycle of approximately 90 days [23,27]. Among others, they are used as the primary avian model for studying song learning, a process that shares similarities with early communication development in various species, including humans [24]. Research by Chen et al. [28], for instance, found that zebra finch song tutors adjust their vocal patterns when teaching juveniles, much like how humans modify their speech when speaking to infants.

Due to their complex social interactions, vocal learning abilities, and cooperation during biparental care, we propose that zebra finches are excellent potential models for investigating social and communicative impairments, including those seen in autism. The similarities between zebra finch vocal learning, i.e., the ability to alter innate vocalizations, and human speech acquisition offer insights into the neurobiological basis of communication disorders [29,30]. Specifically, comparable expression patterns of FOXP1 and FOXP2, two genes associated with coordinated motor control and communication, have been described in relation to vocal learning in both humans and songbirds [31,32,33]. Based on the above, studying the effects of social and environmental factors on zebra finch behavior and neurodevelopment can deepen our understanding of social impairments in humans, potentially leading to better diagnostic tools and therapies for ASD.

Despite the unique opportunity to study a vocal learning system so similar to that of the human [30,34,35], no attempt has been made so far to adapt the VPA model to any of the songbird species. By adapting this well-established research paradigm to a new songbird animal model, the zebra finch, our aim is to offer new insights into the neurodevelopmental mechanisms underlying ASD, potentially contributing to better treatment and intervention strategies by increasing validity. Specifically, we investigated the impact of embryonic (in ovo) VPA treatment on song learning in zebra finches, by focusing on similarities between the songs of male offspring and those of their fathers and non-related other males in the population.

## 2. Results

**Figure 1:**
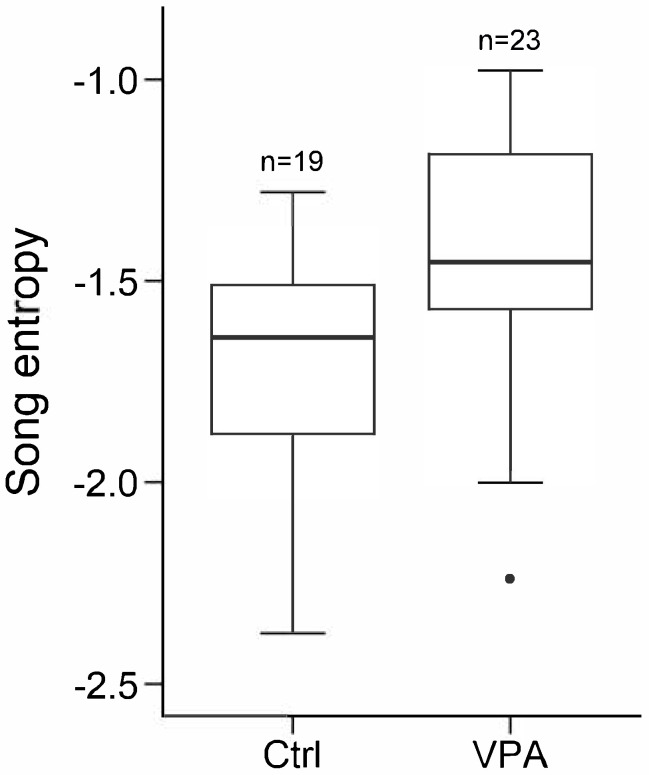
Entropy of female-directed songs of valproic acid-treated (VPA) and control (Ctrl) male zebra finches. Boxplots indicate the median, 1st and 3rd quartiles (boxes), and whiskers represent data within 1.5 IQR from them.

The entropy of female-directed songs produced by VPA-treated males was significantly higher than that of control males (Linear Mixed Model (LMM) of entropy, likelihood ratio tests (LRT) of experimental group: χ^2^_1_ = 8.824, *p* = 0.003; Ctrl → VPA: b ± SE = 0.29 ± 0.09, z = 3.13, *p* = 0.002; Figure 1, Table 1). None of the other investigated acoustic properties showed significant differences between the two groups, although pitch was somewhat (non-significantly) lower in the VPA group than in the control group (LMM of pitch, LRT of experimental group: χ^2^_1_ = 3.131, *p* = 0.077; for all other acoustic properties *p* > 0.542, Table 1).

**Figure 2:**
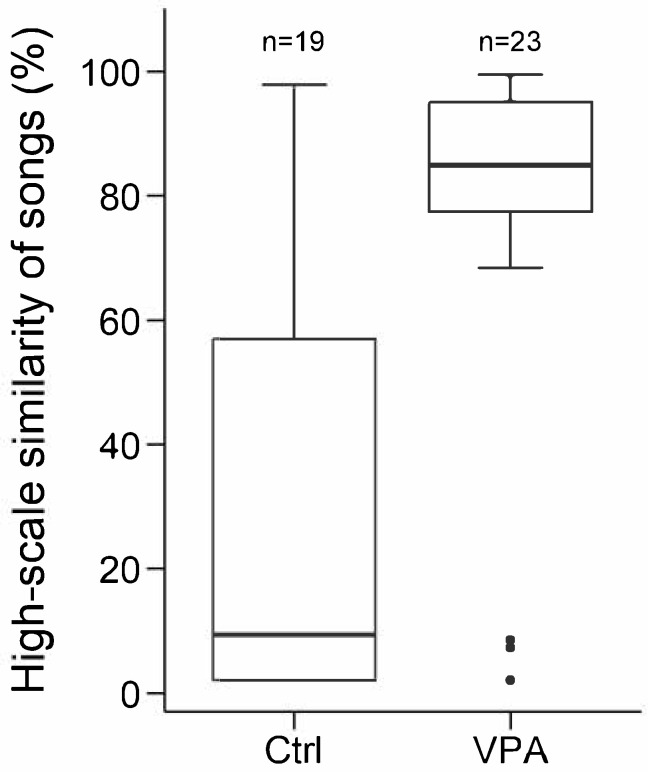
High-scale similarity between female-directed songs of valproic acid-treated (VPA) and control (Ctrl) male zebra finches and their fathers. Boxplots indicate the median, 1st and 3rd quartiles (boxes), and whiskers represent data within 1.5 interquartile range from them.

Male offspring in the VPA group exhibited significantly greater high-scale similarity to their fathers’ songs compared to males in the control group (LMM of high-scale similarity, LRT of experimental group: χ^2^_1_ = 5.720, *p* = 0.017; Ctrl → VPA: b ± SE = 35.04 ± 13.88, z = 2.52, *p* = 0.012; Figure 2, Table 2). Low-scale similarity, sequential match and father–son differences in any of the other investigated acoustic properties were not significantly different between experimental groups (all *p* > 0.144, Table 2). Bold *p* values represent significant differences.

## 3. Discussion

Learning and producing songs are crucial developmental aspects in songbirds and can be paralleled in many ways with human language acquisition and speech. In this study, we demonstrated for the first time that prenatal exposure to valproic acid (VPA) affected male zebra finch offspring’s song learning process, leading to changes in the acoustic properties of the learned song and its resemblance to the father’s song.

A comparison between the songs of fathers and their male offspring revealed that VPA-treated males exhibited a higher syllable-level similarity to their fathers’ songs, indicating that a greater proportion of paternal syllables contributed to their songs. In contrast, control males displayed lower similarity, suggesting that they modified their songs to a higher degree after they were separated from their parents (from post-hatching day 35). These findings imply that VPA-treated males may have difficulties adapting and developing unique song patterns at later developmental stages, potentially due to biological constraints. One possibility is that they are only capable of recalling and reproducing songs heard during early development (up to day 35 post-hatching). Alternatively, they may lack the ability to refine or modify their songs by learning from other males. The song circuitry in zebra finches becomes fully functional by 35 days of age, and a sensitive learning phase occurs between days 25 and 65 post-hatching [36]. During this period, young birds may acquire songs not only from their fathers but also from unrelated males and brothers, after leaving the family [37,38]. In our study, while both VPA-treated and control males initially learned songs from their fathers, results suggest only the control group was able to modify and further develop their vocalizations.

Additionally, VPA-treated males produced songs with higher entropy, suggesting that their vocalizations were more disorganized or closer to random noise as those of control males. Higher entropy usually signifies lower stability and tonality of the song [39]. In general, VPA-treated males retained a more similar song to those of their fathers’ and developed a less organized song compared to controls. A possible explanation for such differences is that early song learning is less affected, whereas crystallization of songs is more impaired by VPA treatment, either by affecting social learning from cage mates or by impairing the self-correcting feedback mechanism underlying song learning.

Our results extend the empirical evidence for prenatal VPA treatment to alter acoustic communication in rodents (mouse and rats) and birds (domestic chicken) [13,40,41,42,43,44]. Although these studies reported prenatal VPA treatment to result in vocalizing at a lower volume and/or with reduced intensity, our results, focusing on the more elaborated vocalization of zebra finches, do not support such an effect. Besides accurately recalling and reproducing a significant portion of their fathers’ syllables, VPA-treated males modulated the amplitude of their songs similarly to control birds. Song complexity in male zebra finches is closely linked to cognitive abilities and plays a crucial role during mate selection. Female zebra finches are attracted to males with intricate and diverse vocalizations [45] and prefer songs with higher frequencies [46]. Additionally, they tend to choose social partners and extra-pair mates based on greater syllable variety in songs [46,47]. Consequently, developing complex, frequent, and powerful songs are under positive selection, as these traits enhance male reproductive success. In contrast, male zebra finches that are not able to refine their songs over time to increase their complexity, individuality, and audibility, develop inferior vocalizations that can reduce their survival and mating success [48].

In conclusion, our results suggest that VPA-treated males could not develop and produce typical, complex songs and we assume an impaired crystallization is behind these findings. Future research should investigate how these VPA-induced changes relate exactly to autism spectrum disorder, i.e., to what extent acoustic communication and/or social impairments (e.g., via impaired social learning) are responsible for such atypical song acquisition and production. To further validate zebra finches as models of vocal development during autism, more experiments are needed. It is imperative to dissect the effects of VPA on the vertical (paternal) and oblique (non-related other conspecifics) influence on the crystallized song. Our study, taken together, suggests VPA-treatment in songbirds, and especially in zebra finches is a promising model to investigate the mechanisms of linguistic impairments associated with ASD.

## 4. Materials and Methods

### 4.1. Subjects

The study was conducted at the Department of Ethology, Eötvös Loránd University, Budapest, Hungary. We used captive zebra finches from the stock population of the Department of Ethology, which was established in 2013 and originated from the domesticated zebra finches maintained at Bielefeld University, Germany [49]. 52 pairs were formed from unrelated individuals, of which 23 pairs reproduced successfully, generating at least one brood; from the resulting 79 offspring 42 were males. Some of the males; therefore, contributed to our dataset with multiple broods (mean ± SD n of broods per male: 1.7 ± 0.9), and some of the broods included more than one male offspring (mean ± SD n of male siblings per brood: 1.6 ± 0.8), and we considered these sources of non-independence in our dataset statistically (see below).

The breeding pairs were housed in separate cages (dimensions: 100 cm × 30 cm × 35 cm), each equipped with a nest box (12 × 12 × 12 cm), attached to the outside of the cage. Coconut fibers were provided as nesting material. Numbered aluminum rings were used to identify individuals (Principle Kft., Újlengyel, Hungary). Room temperature was kept between 18.2 °C and 23.4 °C, and the humidity level between 41% and 66%. An artificial light-dark cycle of 14:10 h was maintained in the room. Birds were provided with ad libitum access to water and food, containing red and white millet (*Panicum miliaceum*), canary grass (*Phalaris canariensis*), and a hint of Niger seed (*Guizotia abyssinica*). To supplement their protein and vitamin intake, the birds received daily portions of egg food (egg food tropical finches, Orlux, Versele-Laga, Deinze, Belgium) and germinated seeds (produced on-site using the aforementioned seed blend). To reduce the stress caused by captivity the animals were kept in the social environment that is natural for their developmental stage (with their family, same sex colonies, or in mating pairs).

### 4.2. Prenatal VPA Treatment

In a separate study (Pogány et al. [50], submitted), we adapted VPA-treatment for zebra finches by optimizing dosage and timing based on offspring survival and development, and using the reported parameters for the domestic chicken as reference [12,51,52]. Briefly, we started from the applied 35 µmol/egg VPA in 200 µL volume per egg in chicken and considered the ca. 60-fold egg mass difference as well as different incubation times between the two species. We then used these derived parameters, and slightly increased/decreased values in a 3 × 3 factorial design (i.e., both dosage and timing had three levels). Based on our findings (Pogány et al. [50], submitted), here we applied 0.45 µmol valproic acid (VPA group) or 0.9% saline solution (control group) on day 9 of incubation; injections had a volume of 3.3 µL per egg.

The first brood of a given pair was assigned to either of two experimental groups randomly, so that all eggs in the brood were injected with either valproic acid solution (VPA) or saline solution (sham control). If there were subsequent broods of the same pair, they were assigned to experimental groups in a balanced design (e.g., if the first brood received VPA-treatment, the second brood received control treatment, and vice versa). Valproic acid solution was prepared by dissolving Sodium valproate (Depakine^®^, Sanofi-Aventis, Paris, France) in sterile, pyrogen-free distilled water. To prevent contamination, alcohol was used to sanitize the needle, the injector, and the egg surface. A sterile needle was used to perforate the eggshell, then the substance was administered into the egg’s air sac using a Hamilton constant rate syringe (CR700, 20 µL volume, Hamilton Central Europe S.R.L., Timișoara, Romania). Following injection, the hole was sealed using a drop of super glue. Eggs were monitored daily until they hatched.

### 4.3. Song Recording and Analysis

**Figure 3:**
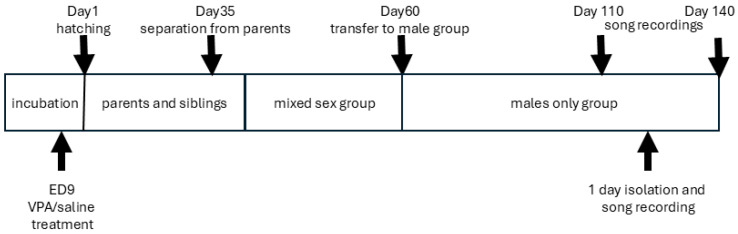
Timeline of the experiment and the social environment of the animals.

Juvenile birds were separated from the parents on day 35 post-hatching (Figure 3). At first, they were housed in (mixed-sex) juvenile cages, and later (around day 60 post-hatching), when their sex-specific traits became evident, subadults were moved into separate male and female aviaries in another room, in which adult males and females were also present [37].

We recorded female-directed songs from 16 fathers and 42 male offspring, including 23 VPA-treated and 19 control males at the age of 133.6 ± 9.2 (mean ± SD) days. One day before the recording, the recorded male and a female stimulus bird were placed into the opposite sides of a double cage within a song recording chamber, separated by a wire mesh barrier. Each father was tested with their current female partner as stimulus, whereas male offspring were tested with randomly selected females. On the following day, female-directed songs were audio and video recorded continuously between 4:00 am and 12:00 am. In case the subject failed to sing at least 10 clear syllables, recording was repeated on a different day; this involved five male offspring (all of these second recordings were successful). We used an Electret Tie-Clip ECM-2500 microphone (Alan Butcher Components Ltd., Blandford, Dorset, UK) and a Mobius Maxi 4K camera (Huizhou Tuopu Xunshi Technology Co., Huizhou, China) for recordings.

We selected the first 10 clearly audible syllables of each subject for song analysis [45] using the Audacity software (v. 3.7.0) [53]. To analyze the selected 10-syllable long songs, we used Sound Analysis Pro 2011 (SAP2011, v.11.104) [54]. The following acoustic properties were calculated and compared between VPA-treated and control male offspring: duration, amplitude, mean frequency, peak frequency, pitch, pitch goodness (an estimate of harmonic pitch periodicity), frequency modulation (FM), amplitude modulation (AM), and Wiener entropy (see Supplementary Materials). Song samples of the male offspring were also compared to those of their fathers based on five acoustic properties: pitch, pitch goodness, FM, AM, and entropy. The automated analysis broke down the samples into larger (50–70 ms) and smaller (5–10 ms) time interval units and provided in percentage how closely the father–son songs resemble each other (high- and low-scale similarities, respectively). Additionally, it calculated the similarity in terms of the sequential structure of the songs (sequential match). Furthermore, we calculated the difference between VPA-treated and control father–son pairs in the following acoustic properties of the songs: pitch, pitch goodness, FM, AM, and entropy.

### 4.4. Statistical Analysis

Statistical analyses were carried out using the R statistical environment (v. 4.4.2) [55]. Each acoustic property was compared between the VPA (n = 23) and control (n = 19) male offspring. Additionally, we investigated differences between the groups in how closely offspring songs resembled those of their fathers and compared the differences in each acoustic property between sons and fathers. Acoustic properties (and father–son differences) were analyzed in separate Linear Mixed Models (LMMs) using Template Model Builder (R package ‘glmmTMB’) [56]. LMMs included the experimental group as a two-level fixed factor (VPA vs. control). In addition, LMMs of acoustic properties included father ID/brood ID as nested random terms, whereas LMMs of father–son acoustic differences included brood ID as a random term to control for non-independence due to multiple broods and siblings from the same father. The effect of experimental group was analyzed by likelihood ratio tests (LRTs), and we provide χ^2^ and *p*-values of LRTs of the models when including or excluding experimental group.

## Figures and Tables

**Table 1:** Linear Mixed Models (LMM) with various acoustic properties of female-directed songs in valproic acid-treated (VPA; n = 23) and control (Ctrl; n = 19) male zebra finches. LMMs were fitted separately for each acoustic property (response), and we provide χ^2^ and *p*-values of likelihood ratio tests between models when including or excluding experimental group as a fixed factor (father ID and brood ID were included in LMMs as nested random terms).

Acoustic Property	Exp. Group	Mean ± SE	χ^2^_1_	*p*
duration (ms)	VPA	90.88 ± 6.41	0.002	0.966
	Ctrl	84.16 ± 8.97		
amplitude	VPA	45.32 ± 0.41	0.372	0.542
	Ctrl	44.57 ± 0.22		
mean frequency (Hz)	VPA	2744.93 ± 66.61	<0.001	0.993
	Ctrl	2743.96 ± 102.21		
peak frequency (Hz)	VPA	2878.36 ± 84.75	0.005	0.946
	Ctrl	2868.75 ± 122.90		
pitch (Hz)	VPA	470.50 ± 35.31	3.131	0.077
	Ctrl	594.27 ± 68.18		
goodness of pitch	VPA	150.04 ± 3.36	0.167	0.683
	Ctrl	146.41 ± 5.64		
FM (kHz)	VPA	42.33 ± 1.06	0.326	0.568
	Ctrl	41.23 ± 1.75		
AM	VPA	0.00 ± 0.01	0.238	0.626
	Ctrl	0.02 ± 0.02		
Wiener entropy	VPA	−1.41 ± 0.06	8.824	**0.003**
	Ctrl	−1.70 ± 0.07		

**Table 2:** Linear Mixed Models (LMM) with father–son differences in various acoustic properties of female-directed songs in valproic acid-treated (VPA; n = 23) and control (Ctrl; n = 19) male zebra finches. LMMs were fitted separately for each acoustic property and difference (response), and we provide χ^2^ and *p*-values of likelihood ratio tests when including or excluding experimental group as a fixed factor (brood ID was included in LMMS as a random term). Bold *p* values represent significant differences.

Acoustic Property (Diff)	Exp. Group	Mean ± SE	χ^2^_1_	*p*
high-scale similarity (%)	VPA	73.63 ± 6.92	5.720	**0.017**
	Ctrl	30.89 ± 8.67		
low-scale similarity (%)	VPA	62.12 ± 4.57	1.501	0.221
	Ctrl	48.60 ± 6.08		
sequential match (%)	VPA	73.20 ± 6.61	2.135	0.144
	Ctrl	87.55 ± 6.78		
pitch difference (Hz)	VPA	1.55 ± 0.18	0.443	0.506
	Ctrl	1.88 ± 0.33		
goodness of pitch difference	VPA	2.91 ± 0.51	0.190	0.663
	Ctrl	2.40 ± 0.71		
FM difference (kHz)	VPA	1.04 ± 0.04	1.433	0.231
	Ctrl	1.14 ± 0.06		
AM difference	VPA	1.10 ± 0.03	1.940	0.164
	Ctrl	0.95 ± 0.06		
Wiener entropy difference	VPA	1.42 ± 0.37	0.351	0.554
	Ctrl	2.03 ± 0.69		

## Data Availability

The data presented in this study is available as Supplementary Materials of this article.

## Supplementary Materials

The following supporting information can be downloaded at: https://www.mdpi.com/article/10.3390/life15071058/s1, Table S1: dataset.

## Abbreviations

The following abbreviations are used in this manuscript:

ASD	Autism spectrum disorder
VPA	Valproic acid
LMM	Linear mixed model
LRT	Likelihood ratio test
